# Modulating mTOR Signaling as a Promising Therapeutic Strategy for Atherosclerosis

**DOI:** 10.3390/ijms23031153

**Published:** 2022-01-21

**Authors:** Anastasia V. Poznyak, Vasily N. Sukhorukov, Alexander Zhuravlev, Nikolay A. Orekhov, Vladislav Kalmykov, Alexander N. Orekhov

**Affiliations:** 1 Skolkovo Innovative Center, Institute for Atherosclerosis Research, Osennyaya Street 4-1-207, 121609 Moscow, Russia; www.fuper@gmail.com; 2 AP Avtsyn Research Institute of Human Morphology, 3 Tsyurupa Street, 117418 Moscow, Russia; vnsukhorukov@gmail.com (V.N.S.); zhuravel17@yandex.ru (A.Z.); xxor2011@gmail.com (V.K.); 3 National Medical Research Center of Cardiology, Institute of Experimental Cardiology, 15A 3-rd Cherepkovskaya Street, 121552 Moscow, Russia; 4 Laboratory of Angiopathology, Institute of General Pathology and Pathophysiology, Russian Academy of Medical Sciences, 125315 Moscow, Russia

**Keywords:** atherosclerosis, mTOR, cardiovascular disease, rapamycin

## Abstract

For more than a decade, atherosclerosis has been one of the leading causes of death in developed countries. The issue of treatment and prevention of the disease is especially acute. Despite the huge amount of basic and clinical research, a significant number of gaps remain in our understanding of the pathogenesis of atherosclerosis, and only their closure will bring us closer to understanding the causes of the disease at the cellular and molecular levels and, accordingly, to the development of an effective treatment. One of the seemingly well-studied elements of atherogenesis is the mTOR signaling pathway. However, more and more new details are still being clarified. Therapeutic strategies associated with rapamycin have worked well in a number of different diseases, and there is every reason to believe that targeting components of the mTOR pathway may pay off in atherosclerosis as well.

## 1. Atherosclerosis

Atherosclerosis is a chronic inflammatory disease that accompanies humans throughout their lives. The processes associated with atherosclerosis originate from endothelium functional modification, which is also characterized by the storage, oxidation, and glycation of low-density lipoprotein (LDL) cholesterol in the inner layer of the arterial wall, as well as the expression of adhesion molecules and chemoattractants [1]. In the intima, where T cells are recruited, monocytes consume oxidized LDL and thus transform into foam cells [2]. The earliest locus of atherosclerosis is fatty streaks that are observed in the aorta during the first 10 years of a human’s life [3]. Fatty streaks transform into atherosclerotic plaques, which contribute to the narrowing of the arteries’ lumen, entailing either ischemic complaints or plaque instability; therefore, the risk of acute atherothrombotic phenomena is quite high [4].

In atherosclerosis, the inflammatory process has an impact on the walls of both large and medium-sized arteries. Today, atherosclerosis is the leading cause of death, and according to statistics, one out of three deaths will be due to atherosclerosis [5]. This disease is caused by unbalanced lipid metabolism and chronic inflammation within the arterial wall. Mechanical or chemical stress leads to endothelial dysfunction, which affects the accumulation of LDL in the intima as well as the recruitment of monocytes and other inflammatory cells [6]. Tissue macrophages inside the arterial wall are transformed into foam cells by assimilation of low-density lipoprotein (LDL) particles [7]. Cell necrosis symbolizes the development of cell damage and the purification of dead cells by macrophages. When efferocytosis in progressive plaques is disturbed, an accumulation of lipids and necrotic debris begins, which results in the appearance of a necrotic nucleus [8]. The growth of plaques is not accompanied by any symptoms; therefore, the inner core turns out to be hypoxic, and the vasa vasorum response leads to neovascularization supplying hypoxic areas. Plaque neovascularization is a significant threat to plaque destabilization and clinical symptoms [9].

The administration of hypolipidemic inhibitors of 3-hydroxy-3-methylglutaryl-coenzyme A (HMG-CoA) reductase (statins) results in significant success in atherosclerosis treatment, thereby decreasing both morbidity and mortality rate [10]. However, the majority of patients gain no advantage from this therapy [11]. New proprotein convertase inhibitors subtilisin/kexin type 9 (PCSK9) can be potentially beneficial for those patients. These drugs are intended exclusively for injection, are present on the market, and are approved by the US Food and Drug Administration and the European Medicines Agency. As an addition to statin therapy, they lower LDL cholesterol levels by 61% and can also significantly lower CVD event frequency [12]. Nevertheless, quite a large number of lipid-lowering drugs have a key role in the treatment of atherosclerosis, since it is impossible to ignore the fact that this disease is inflammatory. Several studies have demonstrated that the suppression of the mechanistic target of rapamycin (mTOR) offers new methods of stabilization; however, there is also a possibility of regression of atherosclerotic plaques [13].

## 2. mTOR Signaling in Health

### mTORC1 and mTORC2

**Figure 1:**
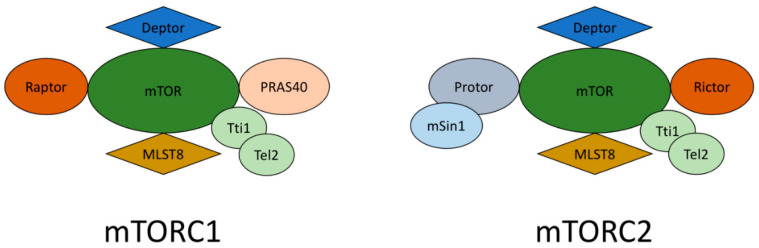
mTORC1 and mTORC2 complexes. This scheme represents a simplified structure of mTORC1 and mTORC2 complexes.

mTORC1 and mTORC2, which are two different multiprotein complexes, contain the highly conserved, constitutively active serine/threonine kinase mTOR; therefore, the cellular functions of these two complexes differ [14]. The schematic structure of mTORC1 and mTORC2 complexes is reflected in Figure 1. As a rule, mTORC1 develops protein synthesis, proliferation, lipogenesis, growth, and energy metabolism. These processes occur with the participation of mTORC1 targets such as S6-kinase 1 (S6K1), 4e-binding protein-1 (4eBP-1), cyclin-dependent kinases (CDKs), and hypoxia-induced factor 1a (HIF1a), which participate in the expression of various glycolytic genes [15]. In addition, mTORC1 participates in the activity and biogenesis of mitochondria. Thus, mTORC1 is highly susceptible to nutrient deficiencies, intracellular energy status, and detected growth factors. The results of the loss of mTORC1 signal transmission are the suppression of cell growth, protein synthesis, and metabolism, as well as the induction of autophagy, the catabolic process of organelle damage, and long-lived proteins aimed at restoring cellular energy levels [16]. It is also known that suppression of mTORC1 favors the longevity of mice, and excessive activation of mTORC1 is linked with different types of cancer [17]. It is also known that mTORC2, which is a less studied type, is in charge of the organization of the cytoskeleton, lipolysis, and insulin susceptibility and is the trigger of several kinases, including protein kinase B (AKT) and kinase 1, which is controlled by serum/glucocorticoids (SGK1) [18]. In male mice, the suppression of mTORC2 results in insulin resistance and shortens life expectancy [19]. In addition, this also results in several imperfections in the polarization of neutrophils and suppresses cell movement, which suppresses the reorganization of the cytoskeleton [20]. Recent studies prove that two mTOR complexes control each other’s actions due to cross-mechanisms [13,21].

Recent studies report that a low concentration of rapamycin is able to suppress mTORC1, while chronic suppression results in mTORC2 inactivation [16]. The RICTOR (rapamycin-insensitive companion of mammalian target of rapamycin) and mSIN1 (mammalian stress-activated protein kinase-interacting protein) relationship is necessary both for maintaining their own stability and for the mTORC2 complex formation, because the elimination of SIN1 overlaps the phosphorylation of AKT in the serine residue 473, which results in a rictor–mTOR infringement [22]. DEPTOR (DEP domain-containing mTOR-interacting protein) is typical for mTORC1 and C2 and operates as an endogenous suppressor for mTORC2 [23]. In addition, mLST8 (mammalian lethal with SEC13 protein 8) is a key component in the mTORC2 complex development, since the elimination of this protein results in mTORC2 imbalance. Despite their uniqueness in many ways, mTORC1 and mTORC2 phosphorylate completely separate substrates and therefore fulfill different functions [24].

mTOR complexes are regulated by several external stimuli, which are nutrients, insulin, growth factors, leptin, and stress signals. At the same time, mTORC1 and C2 react to these factors in various forms and have a special effect on the lower levels. The key effector pathway of mTORC1 is the trigger of ribosomal proteins S6 kinase 1 and 2 (S6K1/2) by phosphorylation of their hydrophobic motif (HM) on Thr389 and Thr388, which results in mRNA biogenesis and the launching of translation and elongation of protein synthesis [16]. Among other mTORC1 substrates, there is 4E (eIF4E)-binding protein 1 (4E-BP1), which also participates in the triggering of gene expression and protein translation. At the same time, the mTORC1 complex is also quite susceptible to various nutrients, especially glucose levels and amino acids. The loss of amino acids, primary leucine, leads to prompt dephosphorylation of S6K1 and 4E BP1 and causes the inactivation of mTORC1 [25]. Similarly, cell energy status is defined by mTORC1, triggered by AMP protein kinase (AMPK). AMPK is phosphorylated in reaction to the reduced energy status of the cell, which shows an elevated AMP/ATP ratio. Activated AMP suppresses cell development by TSC2-dependent inhibition of mTORC1 activity and disables the phosphorylation of S6K1 and 4EBP1 mediated by mTORC1 [13,26,27]. In addition, mTORC1 is also involved in catabolic processes such as apoptosis and autophagy. During fasting, mTORC1 phosphorylates ULK1 (Unc-51-like kinase that activates autophagy). This leads to autophagy suppression due to the prevention of its activation by an essential autophagy activator, AMPK [28].

It is generally recognized that mTORC2 is of great importance in cell proliferation and reacts to development factors such as insulin. mTORC1 acts through various downstream effectors, but mTORC2 usually acts through the insulin/PI3K pathway by phosphorylating AKT in serine residues 473, Thr 308, and Thr 450 when stimulated by insulin [29]. Due to recent investigations, it has become possible to conclude that mTORC2 can phosphorylate AKT in the S377/T479 residues at the C-terminal end and control apoptosis [30]. The mTORC2 subunit of man1 contains a phosphoinositide connecting the PH domain, which plays a key role in the insulin-dependent regulation of mTORC2 activity [31]. The binding of insulin to its tyrosine kinase receptor triggers IRS and recruits triggered PI3K. The phosphorylated hePA 3-PDC pathway acts in an interconnected manner with mTORC2. mTORC2 phosphorylates several protein kinases: A, B, C, G, and SGK1; serum/glucocorticoid-induced kinase 1; and Rho 1 (GDP-GTP-2 exchange protein), which results in their settlement and triggering [32]. With the assistance of RICTOR, mTORC2 straightly phosphorylates the AKT on its Ser473 and promotes the phosphorylation of Thr308 PDK1 (phosphoinositide-dependent kinase 1) as part of the insulin signaling cascade [33].

It is noteworthy that mTORC1 and mTORC2 are constantly in a “dialogue” state, so they are functionally interrelated. MTORC1 likely suppresses mTORC2 by phosphorylation of RICTOR, while mTORC2 controls mTORC1 by phosphorylation of AKT, which controls both AKT activity and AKT profusion [34]. The RICTOR subunit of the mTORC2 complex is capable of phosphorylation by S6K1, the downstream effector of mTORC1, and this phosphorylation adversely controls the mTORC2-dependent phosphorylation of AKT-S473 [35]. On the contrary, when stimulated by development factors, mTORC2 triggers AKT, which increases the activity of mTORC1 by inactivation of TSC1/2 (tuberous sclerosis complex). TSC2 is inactivated by AKT-dependent phosphorylation, which, in turn, destabilizes TSC2 and disrupts its connection with TSC1 [36].

## 3. mTOR Signaling in Atherosclerosis

Several animal studies were conducted by deleting mTOR and stimulating genetic alteration in mTORC1. These investigations revealed mTORC1 involvement in maintaining normal cardiac function. The downstream signaling pathways of mTORC2 within heart cells have not been studied well enough yet. Regardless, there is evidence suggesting that they interact with the Hippo signaling pathway, which is a very conservative signaling mechanism in all organisms responsible for controlling cardiomyocyte proliferation and maintaining heart dimension [37]. Patients suffering from heart failure and experimental animals with diastolic dysfunction differ in the triggering of mTOR and the linked S6K1 signaling mechanism. The most frequent reason for that is the interplay of various factors, such as inflammatory and immune responses, as well as metabolic signaling [38]. mTOR knockout studies and the mTOR/Rheb1 gene deletion studies conducted in animals revealed that both mTORC1 and mTORC2 play a key role in the survival of the embryo and the development of heart cells.

These two studies have shown that mTORC1 is extremely important for the regulation of cardiomyocyte homeostasis and proliferation, protection of cardiomyocytes from apoptosis, cardiac dysfunction, and eventually, cardiac failure. The deletion of the rictor gene, which affects the function of mTORC2, also led to the heart cells anomaly [39]. Therefore, data show that the mTOR signaling pathway plays an important role in the proliferation of cardiac cells. It is important to note that partial inhibition of mTOR shows a cardioprotective effect under specific conditions, such as muscle stress or aging. Since mTORC1 plays an essential role in maintaining the heart’s physiological functions, to guarantee a favorable effect during cardiac stress, only partial suppression of mTORC1 will be needed to slightly suppress mTORC1 crash [40]. Even though mTOR inhibition suppresses myocardial hypertrophy due to mechanical loads in animal models, it has been revealed that overexpression of mTOR lowers cardiac dysfunction as a reaction to hypertrophy caused by pressure overload and lowers the inflammatory response [41].

### 3.1. mTOR Modulation in Atherosclerosis

In 1975, rapamycin was isolated from Streptomyces hygroscopicus on Easter Island. It is a natural macrocyclic lactone with 15 asymmetric centers and 3 conjugated double bonds. Initially, studies were conducted on the antifungal properties of rapamycin, but then, immunosuppressive side effects were also discovered. In the late 1980s, this extremely undesirable effect became an incentive for the development of a clinically valuable drug, sirolimus, and led to widespread studies of the linkage of structural activity (SARs) [42]. In 1993, the first complete synthesis was presented, and later, it was optimized; nevertheless, due to the complexity of the chemical synthesis of rapamycin, it is only of academic interest. To prevent a negative pharmacokinetic profile and reduce immunosuppressive activity, a significant part of SARs was based on a semi-synthetic modification of rapamycin itself. The predominant modifications include the replacement of the hydroxyl residue at the 42-position and the ring-opening reaction [43,44]. Several compounds with a chemical structure similar to that of rapamycin were identified, such as macrolactam FK-506 (tacrolimus, Prograf^®^, Tokyo, Japan), which was received in 1984 from *S. tsukubaensis*, and ascomycin (FK-520), allocated from *S. hygroscopicusyakushimaensis* [45]. These two specific compounds possess powerful immunosuppressive activity and have been subjected to semi-synthetic modifications.

Both rapamycin and rapalogs bind at a low nanomolar level to the FK 506-binding protein (FKBP12—a protein that binds to immunosuppressants) in the FKBP-binding domain, leading to the formation of a binary complex [46]. Through the rapamycin effector domain, the resulting complex binds to the FKBP12–rapamycin-binding domain and induces a conformational modification in the active site of mTORC1, which results in allosteric suppression of the enzymatic activity of mTORC1 [45]. In contrast, mTORC2 is not suppressed by rapalogs. Currently, three rapamycin derivatives are commercially available: temsirolimus (formerly CCI779), everolimus (RAD001), and deforolimus (AP23573). These compounds were produced from a semi-synthetic change of the hydroxyl residue at the 42-position. As a rule, rapamycin is widely used as an immunosuppressant in patients with kidney transplant rejection or as a therapeutic drug for kidney cancer, but it is also popular in cardiovascular medicine [47]. Some data indicate that, in several experimental animal models of atherosclerosis, rapamycin and rapalogs restrain and stabilize the atherosclerotic plaque [13].

Mechanically, the suppression of mTORC1 by rapamycin and paralogs suppresses atherosclerosis by eliminating endothelial dysfunction, suppressing the proliferation and migration of smooth muscle cells, lowering the macrophages’ storage by suppressing monocyte adhesion, and increasing autophagy [48]. Suppression of mTORC1 also contributes to the drainage of cholesterol from macrophages, thereby lowering the foam cells’ number and lipid deposition in plaques [49]. In patients, the risk of cardiovascular disease progression is increased. Due to rapalogs therapy, the frequency of cardiac allograft vasculopathy was reduced. Apart from the positive effects, the use of rapamycin and rapalogs can cause side effects, among which are hyperglycemia, dyslipidemia, and insulin resistance [50]. To achieve a more favorable effect with the administration of rapamycin and paralogs, combination therapy with lipid-lowering statins, PCSK9 inhibitors, and AMPK activators, such as metformin, can be used. In addition, rapamycin can be used as a drug-eluting stent in coronary angioplasty [51]. However, this type of stent has a higher risk of thromboembolic events, although they prevent restenosis. The pro-thrombotic risk is possible to diminish with the use of corresponding drugs, such as Aspirin + P2Y12 inhibitors. This makes drug-eluting stents a better choice in comparison with bare-metal stents [52].

Taking into account the emerging evidence that has revealed the role of autophagy in heart remodeling, rapamycin, as well as its analogs, can be a very powerful therapeutic agent for hypertensive heart diseases. In pulmonary arterial hypertension (PAH), there is a high activation of the mTOR signaling pathway and associated proliferation of smooth muscle cells. The latest research determined that rapamycin-loaded nanoparticles weaken the pathway through the suppressive effects of mTOR [53]. According to this fact, rapamycin also suppresses pulmonary arterial hypertension development induced by TSC1 knockout in smooth muscle cells, weakening the mTORC1 and mTORC2 triggering [54]. As a result of a study of metabolomics conducted in lung smooth muscle cells, it became clear that rapamycin eliminates metabolic disorders in lipogenesis, glutathione, glycosylation, and NAD metabolism in PAH [55]. Further to PAH, it was found that, in animals with cardiac hypertrophy and cardiac failure (caused by transverse narrowing of the aorta), rapamycin weakens cardiac dysfunction and remodeling by suppressing mTOR. Thus, these studies allow us to conclude that targeting mTOR with rapamycin and rapalogs could be a completely new treatment strategy for hypertrophic disease and related heart failure [40].

### 3.2. mTORC1 Inhibition in Atherosclerosis

A real coup in interventional cardiology was the implementation of DES covered with rapalogs [56]. Rapalog-coated DES, in particular, are an upgrade from pure metal stents (BMS), and DES coated with paclitaxel, the proliferation inhibitor, are also an improved version of them [57]. In contrast to rapalogs, paclitaxel suppresses the proliferation of smooth muscle cells (SMCS) by stabilizing microtubule chains [58]. Thus, mTORC1 suppressors perhaps have valuable mechanisms that go beyond the proliferation inhibition in SMCs. Implantation of everolimus-eluting stents in cholesterol-fueled atherosclerotic arteries of rabbits led to notable macrophage purification throughout the plaque without changing the SMC content [59]. This particular impact manifested because plaque macrophages are metabolically very active and therefore extremely susceptible to the suppression of protein synthesis regulated by mTORC1. The clearance of macrophages with everolimus-eluting stents was linked to massive vacuoles development, which is a sign of autophagy. It was also determined that whole-body mTOR knockdown in ApoE−/− mice selectively eliminates macrophages from plaque with the help of autophagy [60]. Since atherosclerosis is an inflammatory process, it is much more meaningful to introduce mTORC1 suppressors systemically rather than as a stent coating. In animal models, well-tolerated rapalogs can be delivered in various ways: orally, intraperitoneally, or subcutaneously using an osmotic mini-pump. A large number of studies on mice with the absence of apoE−/− and low-density lipoprotein (LDL) receptors−/−, similar to rabbits, strive to conclude that the systemic use of rapamycin and everolimus significantly reduces the dimension and complexity of plaques [61]. After 3 years of observation, it turned out that oral usage of rapamycin within the first 14 days after BMS implantation is better received by people; side effects, such as ulcers on the gums or diarrhea, are minimal. It is extremely noteworthy that it has also been demonstrated that this combination is as productive as using DES [13].

As an indirect mTORC1 inhibitor, metformin lowers neointimal development through suppression of SMC proliferation and migration. Another potential mechanism consists of strong autophagy induction, which makes metformin an exclusive antidiabetic drug with confirmed micro- and macrovascular complications lower in patients suffering from diabetes [62].

## 4. Clinical Suitability

Even though rapalogs are well tolerated, the suppression of mTORC1 leads to some adverse effects, which is natural given its central role in various signaling pathways. Some side effects, such as rash, swelling, delayed wound healing, and stomatitis, can manifest both in mild and moderate degrees. More serious consequences such as proteinuria, pneumonitis, thrombocytopenia, and anemia may also occur. These side effects are rare and may become a great limitation in their use [63]. Most of these side effects are potentially directly associated with the use of rapamycin; therefore, these side effects were effectively lowered after the introduction of intermediates with enhanced pharmacokinetic profiles, such as everolimus [64]. In addition, various approaches have been proposed for more successful treatment of these side effects, such as dose reduction and combination therapy. For a more detailed explanation, the following case is given. After the implantation of DES coated with rapalogs, the stopping of proliferation mediated by suppressing mTORC1 suspends the endothelization of the stented vessel, thus elevating the risk of thrombosis [65]. Systemic administration of rapamycin also led to activity growth of the tissue factor. Therefore, double antiplatelet therapy is a common practice after DES implantation.

In addition, metabolic and hemodynamic side effects are possible in the case of rapalog use, and surprisingly, they have an impact on the development of atherosclerosis. Hypercholesterolemia and hypertriglyceridemia are often recorded in patients developing everolimus with transplantation [66]. In atherosclerotic animal models, for example, in ApoE−/− and LDLR−/− mice treated with mTORC1 suppressors, dyslipidemia is also observed [67].

Suppression of the activity of mTORC1 leads to a decrease in the clearance of circulating lipoproteins through suppressing (1) the activity of lipases; (2) the potential to deposit lipids in tissues; and (3) the production of bile acids [68]. There is also evidence of a decrease in hepatic LDL receptors, but the mechanism of the production of bile acids is probably not so significant in dyslipidemia caused by rapalogs, since hypercholesterolemia can appear in mice treated with everolimus [69]. Notwithstanding dyslipidemia, animal atherosclerotic plaques, which are at risk, may decrease in dimension and complexity, and the lipid deposition level in the arteries may lower considerably [70]. Lipid metabolism is regulated by both mTORC1 and mTORC2. mTORC1 contributes to adipogenesis and lipogenesis, while mTORC2 favorably regulates liver lipogenesis and eliminates lipolysis in white adipose tissue [71]. In dyslipidemia mediated by rapalog, the latter can make the suppression of mTORC2 very significant.

According to statistics, between 5 and 32% of patients who take everolimus have hyperglycemia, insulin resistance, and new-onset diabetes mellitus (NODM) [72]. This is why doctors are advised to closely monitor patients receiving rapalogs who have a high risk of NODM. Despite the fact that there is no reliable information about the role of hyperglycemia in cardiovascular diseases yet, it is known that diabetes can accelerate the development of atherosclerosis due to dyslipidemia of endothelial dysfunction and induced inflammation [26]. Additionally, the mTORC1 inhibitor stabilizes endothelial function and lowers the level of inflammatory cytokines.

Excessively increased activation of mTORC1 can lead to insulin resistance due to elevated phosphorylation of the target ribosomal protein mTORC1 S6 (S6rp). On the other hand, S6rp suppresses substrates of the insulin 1/2 receptors (IRS-1/2) and AKT [27]. Caloric restriction leading to mTORC1 suppression and the removal of the target S6rp mTORC1 increases insulin susceptibility [73]. For this reason, it was of particular interest to learn the importance of mTORC1 suppressors in preclinical conditions of diabetes. Acne therapy with a single rapamycin injection, as expected, increases insulin susceptibility and glucose uptake. However, with chronic use, rapamycin surprisingly results in glucose intolerance in both humans and rodents, such as mice and rats [74].

In 2016, a study by Zhao et al. was performed, which showed the safety and effectiveness of the oral administration of low-dose sirolimus in low-density lipoprotein receptor-deficient (LDL r-KO) mice [75].

This effect was partially mediated by the suppression of mTORC2 after chronic administration of rapalog. Recently, it was confirmed that mTORC2 is a critical mediator of insulin susceptibility [76]. Alternative evidence is the resistance of mTORC1 after chronic treatment with rapalog. In vitro experiments with the exploitation of kidney carcinoma cells (RCC) have demonstrated that prolonged treatment with everolimus results in hyperphosphorylation of S6rp, the most important mediator of insulin resistance [77]. As a result, in vivo evidence has been obtained, indicating that chronic mTORC1 suppression in mice treated with everolimus surprisingly resulted in an excessive trigger of mTORC1, as well as a reduction in autophagy (Kurdi et al., unpublished results). Notwithstanding its significant role in the clinic, the ability of paralogs to react differently depending on the duration of their intake or dose is still poorly studied. In antitumor therapy, it is known that chronic administration of rapalogs causes resistance to drugs using various mechanisms [78].

As part of coping with these difficulties, combination therapy was proposed to combat dyslipidemia caused by rapalog and glucose intolerance [79]. The fact that statins and metformin have pleiotropic anti-atherosclerotic effects that go beyond their key action mechanism has long been understood [80,81]. Both drugs induced AMPK in clinically significant doses, which additionally suppressed mTORC1 and triggered autophagy [82]. This can contribute to the reduction in rapalogs dosage and can also exclude several side effects. It is noteworthy that, in patients suffering from diabetes, metformin can lower the LDL level in blood plasma, which enhances its role in the prevention of CVD. Along with drug combinations, to prevent the development of resistance to mTORC1 after chronic administration, a strategy based on rapalogs intermittent dosing regimens can be used, which prevents the development of resistance to mTORC1 after chronic administration. A lower dosage of rapamycin may enhance its selectivity towards mTORC1 and is able to worsen the occurrence of drug resistance or suppression of mTORC2 [83].

## 5. Conclusions

The mTOR signaling pathway plays an important role in the development of atherosclerosis. In addition, it is well known in the context of other diseases, in particular, diseases of the cardiovascular system. A revolution in the field of interventional cardiology was the introduction of DES covered with rapalogs. Unfortunately, the use of rapamycin and rapalogs has many side effects, including hyperglycemia, insulin resistance, and diabetes mellitus (NODM). Based on the available data, the use of drugs that modify the mTOR signaling pathway in the treatment of atherosclerosis is controversial. Some data speak of the unconditional benefits of such a therapy, while the results of several experiments suggest the opposite. Taking into account the undesirable effects, many different regimens, drug combinations, and dosages have been developed. These modifications are bearing fruit, but today, there is no ideal scheme.
